# Potential Medicinal Plants for the Treatment of Dengue Fever and Severe Acute Respiratory Syndrome-Coronavirus

**DOI:** 10.3390/biom11010042

**Published:** 2020-12-30

**Authors:** Mohammed S. M. Saleh, Yusof Kamisah

**Affiliations:** Department of Pharmacology, Faculty of Medicine, Universiti Kebangsaan Malaysia Medical Center, Cheras 56000, Kuala Lumpur, Malaysia; medsaleh@ukm.edu.my

**Keywords:** dengue virus, medicinal plants, ethnobotany, pharmacology, phytochemical, SARS-CoV

## Abstract

While dengue virus (DENV) infection imposes a serious challenge to the survival of humans worldwide, severe acute respiratory syndrome-coronavirus (SARS-CoV) remains the most devastating pandemic in human history. A significant number of studies have shown that plant-derived substances could serve as potential candidates for the development of safe and efficacious remedies for combating these diseases. Different scientific databases were used to source for literature on plants used against these infections. Thirty-five studies described the traditional use of 25 species from 20 families for treating DENV infection with *Carica papaya* and *Euphorbia hirta* were the most widely used across different regions. 13 in vivo studies, 32 in vitro studies, and eight clinical studies were conducted on 30 species from 25 families against different DENV serotypes, while plants from 13 families were reported to inhibit different forms of SARS-CoV, all of which were investigated through in vitro studies. Phytoconstituents belonging to various chemical classes were identified to show a wide range of antiviral activity against these infections. Extensive studies on the potentials of medicinal plants are needed to confirm their efficacy. This paper reveals the capabilities of medicinal plants and their phytochemicals in inhibiting DENV and SARS-CoV infections.

## 1. Introduction

According to the World Health Organization (WHO), infectious diseases remain the sixth leading cause of deaths globally [1]. Emerging and reemerging infectious diseases have continued to be a persistent threat to human population. Among numerous infectious diseases, viral diseases caused by varieties of old and new viruses have imposed a serious challenge to the survival of mankind. Viral infections remain one of the main causes of death worldwide [2]. It is terrifying that the number of patients diagnosed with these infections keeps on increasing every year. Among the top perilous viral diseases are Ebola (over 11,000 deaths in 2019) [3], acquired immunodeficiency syndrome (AIDS) which accounted the deaths of 1.1 million people globally [4], influenza (300,000–500,000 deaths yearly) [5,6], dengue fever (over 390 million, of which 96 million manifest clinically) [7], and severe acute respiratory syndrome coronavirus 2 (SARS-CoV-2) (also known as COVID-19 in the 2019 outbreak) (over 60 million cases with approximately 1,416,292 deaths worldwide) [1].

With the emergence of new viral strains and increasing resistance to standard antiviral therapy, and the fact that most of the available antiviral therapies are non-specific for particular viruses [8], the key focus of medical research is therefore centered on the development of specific and cost-effective antiviral regimens [9,10], which include alternative treatment from herbal sources. At present, several antiviral vaccinations are available including human papilloma virus [11], hepatitis A and B virus [12], varicella, measles, mumps, rubella [13], and rabies [14]. However, there are still no vaccines for a few other viral infections.

**Figure 1:**
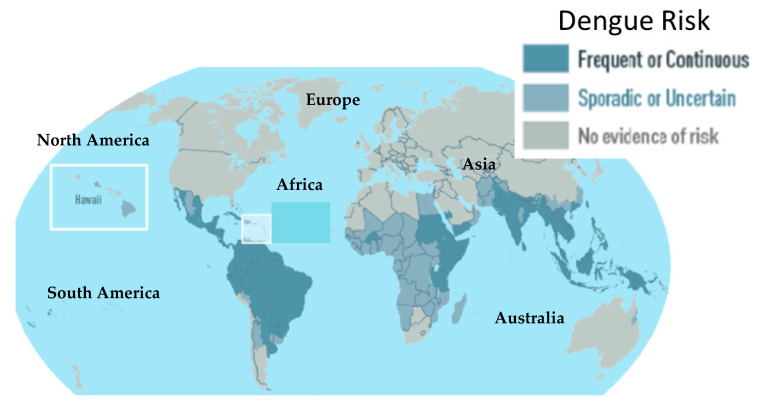
Global map showing the regions affected by dengue. Adapted from [28].

Dengue fever is a viral infection that has imposed serious economic burdens in both tropical and sub-tropical regions [15]. More than 300 million cases are recorded annually with nearly 22,000 deaths [16]. Globally, dengue fever affects approximately 2.5 billion people with an estimated 50–100 million new cases annually [17]. The endemic disease occurs in more than 100 countries including Africa, Asia, America, Eastern Mediterranean and Western Pacific being the most affected regions [18] (Figure 1). Recently, the occurrence of dengue has rapidly increased, reaching the pandemic level [19]. More than 20 African countries were affected by an epidemic between 1960 and 2017. However, many outbreaks are never officially reported [20]. Dengue fever is caused by four distinct but antigenically related flavivirus serotypes with dengue virus (DENV) type-2 (DENV-2) reported to be more deadly than all other serotypes [21]. The disease is prevalent in urban and semi-urban areas and is transmitted by female *Aedes aegypti* mosquitoes [22]. The incubation period after the virus gets into the human body varies from 3–14 days, after which symptoms ranging from mild to severe begin to manifest. Early symptoms may include headache, fever, severe muscle, and joint pain [23]. Severe cases are characterized by hemorrhagic fever syndrome, which may result in dengue shock syndrome [24]. Its treatment includes standard management of general fever as well as other symptomatic treatments [25]. Dengvaxia, the first dengue vaccine, was licensed a few years back for clinical use [26]. The safety of the vaccine however, is still questionable due to findings that it could increase the risk of dengue fever severity in individuals with no prior history of DENV infection [27].

On the other hand, the novel COVID-19 is currently a global health crisis threatening the whole world. It has swiftly spread across various countries from its discovery in Wuhan City, Hubei Province of China [29]. As of 9 December 2020, over 60 million cases of COVID-19 and 1,416,292 deaths have been reported [1]. There have been two incidents of transmission from animal β-coronaviruses to humans in the last two decades which had resulted in serious diseases [30]: severe acute respiratory syndrome coronavirus (SARS-CoV), which was passed from bats onto humans through palm civet cats (intermediary host) in China in 2002–2003 [31], and Middle East respiratory syndrome coronavirus (MERS-CoV), transmitted from bats to humans via dromedary camels in 2012 in Saudi Arabia [32]. The latest coronavirus outbreak, recognized as SARS-CoV-2 or COVID-19, occurred in December 2019. It has more than 95% homology and more than 70% similarity with the bat coronavirus SARS-CoV-2 HKG/HKU strain and SARS-CoV, respectively [30]. Since then, the number of cases keeps increasing rapidly with the majority of human-to-human transmission [33]. As of 9 December 2020, the United States has the highest number with almost 15 million cases and more than 281,000 deaths, followed by India (almost 10 million cases and 141,360 deaths) and Brazil (six million cases and almost 178,009 deaths) [34]. Prevention of this disease is very crucial since there is no approved treatment for the infection. However, few vaccines have been developed for COVID-19 [11] with the hope that the pandemic could be lessened and finally eradicated.

Although DENV and SARS-CoV belong to different viral families and their characteristics such as structure, entry mechanism, replication, and pathogenicity are different, they manifest similar early signs and symptoms of fever, headache, joint pain, low platelet and white cell counts [35], which could lead to misdiagnosis. Both DENV and SARS-CoV are endemic to several parts of the world, while COVID-19 has become a pandemic. More importantly, some medicinal plants and their bioactive compounds have been reported to be effective for the inhibition of both infections, either by reducing viral load or by preventing viral replication. *Houttuynia cordata* and *Boesenbergia rotunda* are such examples. The extracts of both plants have been reported to inhibit both DENV-2 [36,37] and SARS CoV-2 [38,39,40]. Furthermore, compounds such panduratin and quercetin from these plants and other plants also inhibit both viral infections.

A greater percentage of people in tropical and sub-tropical regions rely on herbal medicine to cure various diseases and infections. Therefore, natural products could be one of the major sources for the development of antiviral drugs. About 80% of the population in developing countries, especially in Asia and Africa, use natural products from plants for their primary healthcare [41]. Plant extracts from different plant parts (stem, root, leaves, seeds, fruits, and flowers) [42], phytoconstituents (isolated compounds), nutraceuticals, and dietary supplements have been extensively used in treating a wide range of infectious and non-infectious diseases. They have also become the main sources of tested material in the development of several drugs including antiviral drugs based on ethnomedicinal practices [43]. According to the WHO, 25% of the commonly used drugs contain compounds that are purified from plants [44]. Due to many harmful side effects of synthetic drugs and increased microorganism resistance to standard antimicrobial therapy, alternative treatments from traditional herbal medicines have been widely explored [45]. Several medicinal plants have been reported to possess significant antiviral properties at different stages of viral growth [46,47] and many have been used for treating viral infections, both in humans and animals including DENV infection [48,49,50]. Moreover, many phytoconstituents have been suggested to be potential inhibitors of SARS-CoV through in silico studies [51] and molecular docking/modeling [52,53,54]. The aim of this article is therefore to provide a concise review on the potential medicinal plants for the treatment of DENV infection and SARS-CoV by providing more insights on the role of natural remedies such as medicinal plants and pure bioactive metabolites on the development and advancement of anti-DENV and anti-SARS-CoV drugs.

## 2. Evidence Acquisition

A systematic literature search using Google Scholar, Scielo, Scopus, Web of Science, and PubMed was conducted to determine various medicinal plants traditionally used for treating DENV and SARS-CoV infections from May to September 2020. Sets of keywords were used for the search. The keywords for DENV were “traditional plants” AND “dengue”, “active plant extracts” AND “dengue and bioactive compounds” AND “dengue”; while for SARS-CoV they were “medicinal plants” AND “SARS-CoV” and “bioactive compounds” AND “SARS-CoV”. A total of 96 full text articles were retrieved on DENV, and 42 on SARS-CoV infections were included in this review.

## 3. Traditional Plants Used for Dengue Treatment

Many plants from the Lamiaceae family are used for the treatment of DENV infection by various tribes across different regions. However, the most commonly used plants are *Carica papaya* (Caricaceae) (papaya) and *Euphorbia hirta* (Euphorbiaceae), as shown in Table 1. Different parts of these plants such as leaves, roots, stems, and flowers are used in different formulations—decoction, infusion and leaf juice—to treat the ailment. In the Philippines, papaya leaves are pounded and squeezed to extract the juice, which is then orally administered to affected patients to improve low platelet count [55,56]. In Fiji, a decoction of the female papaya leaves is used [57]. Mixture of the fruits and leaves are used by the people of Tamil Nadu in South India to improve the symptoms of dengue fever [58], while Lawachara Forest Reserve dwellers in Bangladesh use an oral decoction of unripe fruits for the same purpose [59]. Meanwhile, the use of different parts of *Euphorbia hirta* is reported to cure dengue fever. Some use a water decoction of the leaves and stems [56,60,61], while topical uses of stem barks, barks and roots are also reported to reduce dengue fever-related symptoms like fever, headache, skin blister, nose and mouth bleeding [61]. Laguna villagers in the Philippines use a decoction of the roots, stem, and leaves of the plant [55]. Other notable plants that are traditionally utilized for DENV treatment include leaf juice of *Mikania cordata* (Asteraceae) used by the Santal tribe in the Thakurgaon District of Bangladesh to bring down the fever [62], and a decoction of barks and bulbs of *Annana reticulata* and the leaves of *Mentha arvensis* (Lamiaceae), *Synsepalum dulcificum* (Sapotaceae) and *Vitex negundo* (Lamiaceae) used by the Matigsalug tribe in Davao City, the Philippines [60]. Additionally, decoction of *Protium spruceanum* (Burceraceae) stem bark with lemon was also reported by the Porvenir community in India to effectively cure dengue fever [63], whereas its Chhattisgarh tribes use the leaf extract of *Azardirachta indica* (Meliaceae) [64]. Decoction of a mixture of the rhizome and leaves of *Zingiber purpureum* (Zingiberaceae) with a clove of onion and turmeric are used by the Atingola people of Indonesia for similar treatment [65].

## 4. Pharmacologically Active Medicinal Plants and Isolated Compounds against Dengue Virus

After comprehensive search on databases, 13 in vivo studies, 32 in vitro studies, and eight clinical studies were identified to have been conducted on 30 plant species from 25 families against DENV infection. As previously mentioned, *Carica papaya* and *Euphorbia hirta* are the most popular species in traditional herbal medicines for treating DENV infection. The most reported plant part investigated is the leaves.

### 4.1. In Vivo Studies

One of the significant clinical features of dengue fever is a reduction in platelet count. Many plant species have the ability to boost platelet count. Leaf extract of *C. papaya* has been demonstrated to augment platelet count in various dengue fever models in animals [76,77,78], in addition to reducing clotting time in thrombocytopenic rats [76]. Other plant species that show similar activity are *Ipomea batata* and *Aternanthera sessillis* [77], *Eurycoma longifolia* [69], and *E. hirta* [15,77,78]. However, *M. charantia* did not show similar effects in the DENV-infected Sprague-Dawley rats [77]. Aqueous extract of *Eurycoma longifolia* root also showed 30% reduction in viral load in AG129 DENV-infected mice [79]. Another study demonstrated that oral administration of *Curcuma longa* remarkably reduced viral loads in ddY mice after 24 h [80]. Aqueous extract of *Azadirachta indica* leaves at 20–30 mg/mL (maximum non-toxic concentration) completely inhibited viral replication as confirmed by the absence of virus specific 511 bp amplicon in real-time polymerase chain reaction (RT-PCR) and the absence of DENV-related symptoms in DENV-infected suckling mice [81]. Freeze-dried *C. papaya* leaves also increased plasma monocyte chemoattractant protein-1 (MCP-1) levels during the peak of viremia when given orally to AG129 dengue-infected mice, suggesting the possible immunomodulatory capacity of this plant during DENV infection [82].

It seems that *C. papaya* is the most studied plant in the dengue fever model. However, more research studies need to be conducted to better understand the beneficial effects of the plant extracts and the active phytoconstituents on DENV infection biology. The effects of medicinal plants on the pathophysiology of dengue concerning the mechanisms that govern the causes of capillary leakage and decrease in platelet count could also be studied in animal models. Many mouse models such as knockout mice or transgenic mice can also be employed to evaluate anti-DENV activity of the medicinal plans [83]. Therefore, any involvement or modulation of certain genes by the extract could be investigated.

### 4.2. In Vitro Studies

Virtually all in vitro studies were centered on the inhibitory effects of various medicinal plants against DENV replications. Infection by cytocidal viruses is usually associated with changes in cell morphology and physiology as well as sequential cellular biosynthetic events. Reduction in cytopathic effect—structural changes in host cell due to viral invasion—is the major technique used for the detection of the extract’s inhibitory capacity against DENV. Polar extracts such as aqueous [36,81,84], methanol [50,84,85,86], and ethanol [87,88] have been reported to possess anti-dengue activity. The inhibitory potential is attributable to the presence of polyphenols and terpenes [88]. The extract showed good inhibitory effects that ranged from 0.8 µg/mL to 1900 µg/mL with percentage inhibition more than 34% up to total inhibition. Methanol extracts of *Cymbopogan citratus*, *Andrographis paniculata*, *Momordica charantia*, *Ocimum sanctum*, *Pelargonium citrosum*, and *Citrus limon* showed inhibitory effects on DENV type-1 (DENV-1) growth in Vero E6 cells with *A. paniculata* exhibiting the highest activity followed by *M. charantia* [50]. Similar findings were also reported in ethanol and aqueous extracts of *A. paniculata* leaves [84,85]. The ethanol extract possessed higher inhibitory potential (75%), attributable to the presence of polyphenols and terpenes [88]. Meanwhile, another study confirmed the strong inhibitory effect of a *Cymbopogon citratus* methanol extract of about 98.8% against DENV-2 New Guinea C strain (NGC) at a dose of 20 µg/mL [86]. Other extracts that showed similar inhibition include *Acorus calamus* and *Myristica factual* (96.5% and 122.7%, respectively). Aqueous extract of *Azadirachta indica* leaves had totally inhibited 100–10,000 50% tissue culture infective dose (TCID_50_) of DENV-2 at its maximum non-toxic concentration (1.897 mg/mL), marked by the lack of cytopathic effects [81]. In a similar context, leaf methanol extracts of *Spondias mombin* and *Spondias tuberosa* showed promising inhibitory capacity against DENV-2 replication at concentrations of 3.31 and 17.98 µg/mL, respectively, believed to be due to the presence of rutin, quercetin, and ellagic acid in both plants [85]. Petroleum ether extract of *Alternanthera philoxeroides* was also reported to strongly inhibit DENV with a median effective concentration (EC_50_) value of 47.43 µg/mL [89]. Other plants that were reported with the activity include *Rhizophora apiculate*, *Flagellaria indica*, and *Cladogynos orientalis*, *Houttuynia cordata* [87], *E. hirta* [90], C*issampelos pareira* [82], *Curcuma longa* [72], and *Psidium quajava* [83].

Almost all parts of the above-mentioned plants like aerial parts [82], roots [83], and stem barks [83,84] as well as the whole plants [85] show protective effects against DENV, which are not restricted to DENV-2 or DENV-1 only, but also against type 3 (DENV-3) and type 4 (DENV-4) [82,83]. Various medicinal plants investigated in vitro, in vivo, and clinical studies against DENV infection are shown in Table 2. Generally, many medicinal plants extracted mostly with polar solvents such as water, methanol, and ethanol exhibit significant antiviral activity. We could therefore infer that these solvents are capable of extracting the antiviral compounds from any medicinal plants. Mechanistic antiviral studies of the plant extracts are still lacking. More studies need to be conducted to explore this aspect to combat DENV infection.

### 4.3. Clinical Studies

Among many medicinal plants with anti-DENV potentials, *Carica papaya* has been tested clinically in many studies. Administration of 25 mL of plant aqueous leaf extract was found to reduce the severity of dengue fever in patients by increasing platelet, white blood cell as well as neutrophil counts in the blood [91]. Similarly, in a clinical study conducted on 12 patients with dengue fever with a platelet count less than 130,000/mm^3^, two doses (5 mL) of *C. papaya* leaf extract administered at 8 h intervals along with standard dengue symptomatic care, resulted in an increase in platelet count and white blood cell count within 24 h of treatment [92]. Oral administration of *Carica papaya* leaf juice at a dose of 30 mL for three consecutive days also elevated mean platelet count in 228 patients with dengue fever and dengue hemorrhagic fever compared to the control group [93]. It therefore could prevent the complications of thrombocytopenia [94], and shorten the length of hospital stay in these patients [95]. Similar outcomes were also reported by a recent study [96] that also showed *C. papaya* treatment reduced plasma tumor necrosis factor-α, interferon-γ, and interleukin-6 as well as mean dengue non-structural protein 1 (NS1). The findings confirmed the immunomodulatory property of the plant seen in the animal study. A clinical study on *E. hirta* aqueous extract demonstrated that oral administration of the extract to patients with dengue fever raised platelet and total leukocyte counts, but had no significant effect on hematocrit [97]. Not many clinical studies on the anti-DENV activity in medicinal plants have been carried out. More such studies are needed to confirm the effectiveness of the extracts and/or their isolated bioactive compounds on DENV.

### 4.4. Anti-Dengue Bioactive Components from Medicinal Plants

The bioactive constituents reported here showed different degrees of inhibitory activities against different serotypes of DENV. The isolated constituents belong to various chemical classes such as glycosylated and non-glycosylated polyphenols including flavonoids, sulfated polysaccharides, alkaloids, quinones, and terpenoids. Antiviral activity of andrographolide, a diterpene isolated from *Andrographis paniculata* showed significant inhibitory activity against DENV-2 in HepG2 and HeLa cell lines with EC_50_ of 21.304 and 22.739 µM, respectively [118]. The compound also possessed larvicidal activity, which effectively increased the percentage of *A. aegypti* larvae mortality, concentration-dependently [119]. This property might be useful for the management of dengue vector.

A total of 51 bioactive compounds obtained from various plant sources were reported in this article to exhibit different degrees of anti-dengue activity. These compounds comprise six terpenoids, four phenolic acids, two alkaloids, a saponin, seven polysaccharides, 14 flavonoids, and 17 phenolics and flavonoid glycosides and derivatives. Plaque- and focus-forming assays used in in vitro studies are the major techniques for determining anti-dengue activity of potential compounds. Both assays are run to quantify the viral titer. The former is used to ascertain the number of DENV infectious particles (virions) by the means of plaque formation, while the latter is a variation of the former assay, which employs an immunostaining technique. The assay quantifies infected host cells and infectious DENV virions before the formation of a plaque after DENV-induced cell lysis.

Castanospermine, an alkaloid derived from black bean (*Castanospermine australe*), was investigated against all serotypes in vitro via plaque-forming assay using BHK-21 cells and in vivo using A/J mice. The compound inhibited all serotype infections in vitro at the level of secretion and infectivity, but could only inhibit DENV-2 in vivo and prevented mouse mortality [120]. These findings suggest that not all compounds that are active in in vitro studies would give similar outcomes in in vivo studies, more so in clinical studies. The percentage of inhibitory activity of these compounds ranged from 20–70%, with phenolics and flavonoid glycosides and derivatives exhibiting the highest activity. Many of the compounds are selective in their anti-dengue activity against different serotypes. Most of them displayed activity against DENV-2. Quercetin and quercetirin obtained from the ethyl acetate fraction of *H. cordata* aerial leaves displayed anti-DENV-2 effects with median inhibitory concentration (IC_50_) of 176.76 µg/mL and 467.27 µg/mL, respectively, showing a bigger virucidal effect of quercetin than its glycoside, quercetirin. However, the quercetin was more cytotoxic (CC_50_ = 155.38 µg/mL) than the quercetirin [108]. In another study, four constituents from the ethyl acetate fraction of the same plant—chlorogenic acid, hyperoside, quercetin, and quercetirin—were tested against the NGC strain of DENV-2 using the plaque reduction assay individually displayed certain degrees of activity, while their combination yielded greater antiviral effects [109]. The main phenolic constituents of *Spondias mombin* and *Spondias tuberosa*—rutin, quercetin and ellagic acid—showed antiviral effects at 500 µg/mL and inhibited viral replication by 68.42%, 50%, and 25.02%, respectively in C6/36 cells. Rutin was the most active [85]. Among the compounds purified from the methanol extract of yellow rhizomes of *Boesenbergia rotunda* and tested against DENV-2, four compounds exhibited anti-DENV activity. These compounds were chalcone derivatives: 4-hydroxypanduratin A, panduratin A, pinostrobin, and cardamonin. The first two compounds exhibited good competitive inhibitory activity toward DENV NS3 protease, while the last two compounds demonstrated non-competitive inhibitory activity [37].

Fucoidan, a sulfated polysaccharide isolated from *Cladosiphon okamuranus* (marine alga) inhibited DENV-2 infection tested in BHK-21 cells using the focus-forming assay. The compound reduced infectivity by 20% at a concentration of 10 µg/mL. However, based on the structure–activity relationship, conversion of glucuronic acid—a residue present in the fucoidan—to glucose resulted in the loss of the compound inhibitory activity, suggesting its critical involvement in fucoidan antiviral function, whereas removal of the sulfated functional group resulted in the attenuation of DENV-2 infection similar to that of fucoidan (21%). This suggests that the sulfated group does not have any significant effect on antiviral activity against DENV-2 infection [121]. Another sulfated polysaccharide, galactans isolated from *Cryptomenia crenulata* [122] and *Gymnogongrus tolulosus* [123], were reported to inhibit DENV-2 replication in Vero cells with IC_50_ of 1.0 µg/mL and 0.19–1.7 µg/mL, respectively. Galactan obtained from *C. crenulata* has selective activity. The compound was reported to be totally inactive against DENV-1, but exhibited good inhibitory effects against DENV-2 and DENV-3, but displayed weak effect on DENV-4 [122], where the protective effects could be via inhibition of the virus adsorption or virus internalization [124]. Another isolated sulfated polysaccharide called kappa-carrageenan from *Gymnogongrus griffithsiae* selectively inhibited DENV-2 replication (IC_50_ = 0.9 µg/mL), but exhibited lower effects against DENV-3, and was totally inactive against DENV-1 and DENV-4. This compound was also obtained from *Meristiella gelidium* and exhibited anti-DENV-2 activity with IC_50_ between 0.14–1.6 µg/mL [125]. The findings suggest that both sulfated polysaccharides have the potential to be developed as anti-DENV-2 and anti-DENV-3 drugs.

**Figure 2:**
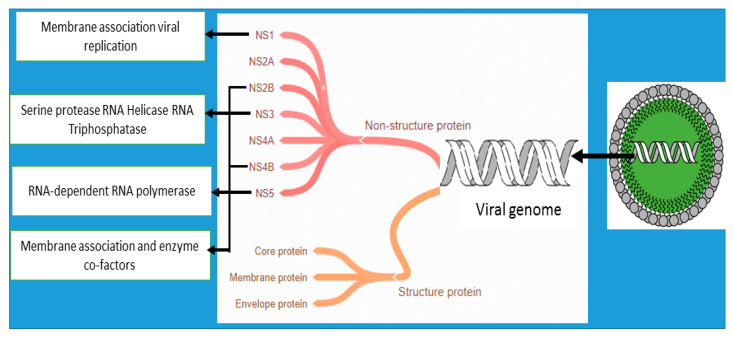
Diagrammatic representation of possible inhibitory sites against DENV.

Many other compounds isolated from various plants that display anti-DENV activities are shown in Table 3. To date, antiviral agents for DENV that have been developed act by inhibiting viral entry, RNA-dependent RNA polymerase (RdRp), protease, or helicase enzymes (Figure 2) [126]. So far, the plant compounds purified from trunk bark of *Crypotocarya chartacea*, mono- and dialkylated flavanones (chartaceones 1–6) have shown remarkable non-structural protein 5 (NS5) RdRp inhibitory activity with IC_50_ ranging from 1.8 to 4.2 µM [127]. Purified daphane diterpenoid orthoesters from the barks and woods of *Trigonostemon cherrieri*—trigocherrins A and trigocherriolides B and C—also inhibited DENV RdRp, while no effect was seen with trigocherrins B and F as well as trigocherriolide A [128]. No plant compounds with anti-DENV property that could act via helicase and protease inhibitory activities have been reported. Most of the studies are still at the stage of activity screening. More works need to be done to explore the anti-DENV potential with their mechanism such as the inhibition of viral genomes and the destruction of enzymes necessary for viral encoding to find potential candidates for DENV treatment.

Another aspect that could be investigated is the indirect effects of these compounds on the DENV biology. Studies have shown the involvement of oxidative stress and inflammation in DENV infectivity [137,138]. Most of the bioactive compounds that have anti-DENV activities are polyphenols and diterpenoids. These compounds have been abundantly reported to possess antioxidant and anti-inflammatory activities in many studies [139,140,141]. However, investigation of the medicinal plant antioxidant and anti-inflammatory effects on dengue fever pathogenesis is still lacking. Only a study by Tseng et al. [93] have studied this aspect. They reported that andrographolide induced heme oxygenase-1 (HO-1) activity, an antioxidant enzyme in mice that strongly delayed the disease onset and mortality by activating the antiviral host type-1 interferon (IFN) response in an infected mice model as well as reduced DENV-1–4 loads in the infected mouse brains [130]. HO-1 is an enzyme that catalyzes the conversion of heme into carbon monoxide, biliverdin, and iron. It is known that HO-1 exhibits its cytoprotective effects through antioxidant effects [142,143]. This suggests that any therapy or drug capable of inducing the HO-1 signaling pathway could be a promising candidate of treating DENV infection.

## 5. Potential Traditional Plants-Based Medicines for SARS-CoV-2 (COVID-19)

For years, natural products from plant-based origin have been an indispensable source of medicinally active constituents that are essential for drug discovery and development for the treatment of numerous diseases. Many herbal medicines have been recognized to play a tremendous role in the treatment of emerging pandemics, specifically the ability to block the SARS virus from binding to its receptor, angiotensin-converting enzyme 2 (ACE-2) [144]. Increased ACE-2 activity and expression have been known to modulate the inflammatory cytokine levels, most notably interleukin-1 and high-mobility group box 1 (HMGB1). The presence of the glycan-mimicking structure in most bioactive compounds (especially phenolic compounds) makes them suitable for various protein-inhibitory roles [145]. One of the plant-based natural products is a traditional Chinese medicine approved for the treatment of the SARS-CoV outbreak in 2003 [146], leading to the employment of traditional Chinese medicine in the current COVID-19 treatment in China [147]. Furthermore, various herbal plants from different families have been investigated and proven to be efficacious in the treatment of this pandemic (Table 4). Water extract of the whole plant of *Houttuynia coedata* was reported to slow down viral replication through pivotal enzyme inhibition against SARS-CoV as a result of its ability to inhibit 3C-like protease (3CL^pro^) and RdRp of the virus [39]. Similar water extract of young leaves of *Toona sinensis* also exhibited good inhibitory effect against SARS-CoV and human coronavirus 229E (HCoV 229E) replication [148]. Anti-SARS-CoV activities of several other plant extracts such as ethanol extract of *Euphoria nerifolia* leaves [149], polar and non-polar extracts of Chinese’s medicinal herbs such as *Cibotium barometz*, *Gentiana scabra*, *Dioscorea batatas*, *Cassia tora*, and *Taxillus chinensis* [150], water extract of *Istatis indigotica* root [151], and ethyl acetate soluble fraction of *Angelica keiskei* [152] were also reported. Thus far, neither in vivo nor clinical studies involving herbal medicines and SARS-CoV have been reported.

Phenolic compounds, especially flavonoids, have been identified as suitable candidates for binding and inhibiting ACE-2 and SARS-CoV-2 S protein through in silico crystal structure modeling [155]. Quercetin obtained from different plant sources has been reported in different studies to inhibit SARS-CoV-related infection. Its purified compound from the tender leaf extract of *Toona sinensis* was reported to inhibit cellular entry of SARS-CoV and prevent viral replication [148]. Luteolin from Chinese herbs exhibits significant anti-SARS-CoV activity with 50% efficacy at a concentration of 4.5 µM [156]. Scutellarein, obtained from *Scutettaria baicalensis* and *Asplenium belangeri* as well as myricetin from *Myrica rubra* inhibit the ATPase activity of SARS-CoV helicase nsP13 [157]. Inhibition of this enzyme is another therapeutic option for coronaviruses because it results in inhibition of viral replication [158].

Trans-(3,5,4′-trihydroxystilbene), a major compound present in abundance in *Polygonum cuspidatum*, *Vitis vinifera*, and *Vaccinium macrocarpon*, which exhibits inhibitory activity against MERS-CoV infection [159]. Other polyphenols with inhibitory activity against SARS-CoV include gallocatechin gallate, procyanidin B1 [154], and kaempferol [153], which have been investigated in vitro and proven to effectively treat SARS-CoV and MERS-CoV infections. Other compounds reported with anti-SARS-CoV (Table 5) include an alkaloid called lycorine purified from *Lycoris radiate* [160], glycyrrhizin, a saponin isolated from the root of *Glycyrrhiza glabra* [161], tetra-O-galloyl-ß-D-glucose, the major component of *Galla chinensis* [156], sinigrin, indigo emodin, and hesperetin from water extract of *Istatis indigotica* [151]. Emodin from *Rheum* and *Polygonum* genera blocks the SARS-CoV spike protein and ACE interaction in order to prevent SARS-CoV invasion [162] and inhibits SARS-associated coronavirus 3a protein [163].

Among the antiviral effects of the plant-based bioactive compounds are papain-like protease (PL^pro^) and 3CL^pro^ inhibitions. Both proteases are crucial in the viral life cycle. Therefore, inhibition of these proteases observed in many medicinal plants would then curb the viral replication. 3CL^pro^ is a major protease in coronavirus. It is involved in catalytic cleavage of the viral polyproteins at 11 cleave sites, producing 12 individual viral proteins which then form functional complexes essential in viral replication [164]. Biflavonoids like amentoflavone, apigenin, and luteolin, purified from *Torreya nucifera*, inhibit 3CL^pro^ function [165]. Polyphenols purified from the most active chloroform fraction of *Broussonetia papyrifera* roots selectively exhibit higher inhibitory activity against PL^pro^ than 3CL^pro^, and papyriflavonol is among the compounds that has the highest inhibitory effects on PL^pro^ with an IC_50_ value of 3.7 µM [153]. Xanthoangelol, an alkylated chalcone isolated from the ethyl acetate soluble fraction of *Angelica keiskei* leaves, is also active against both 3CL^pro^ and PL^pro^ with IC_50_ values of 11.4 and 1.2 µM, respectively [152]. Diarylheptanoid compounds isolated from the ethanol extract of *Alnus japonica* barks also exhibited dose-dependent inhibitory effects against PL^pro^. Among the compounds, hirsutenone showed the highest effect with IC_50_ of 4.1 µM [166]. In another study, sinigrin, indigo emodin, and hesperetin isolated from the water extract of *Istatis indigotica* inhibited 3CL^pro^ of SARS-CoV via cell-based cleavage and cell-free cleavage assays [151]. Cell-free cleavage assay is an enzyme immunoassay to detect anti-3CL^pro^ effect by the plant extract. In this assay, viral purified recombinant 3CL^pro^ expressed in *E. coli* is incubated with a substrate fusion protein (cleavage substrate) and plant extract before the detection of non-cleavage substrate protein using enzyme immunoassay. On the other hand, purification of 3CL^pro^ is not necessary in a cell-based assay. Plasmid that carries 3CL^pro^, cleavage substrate, and luciferase is co-transfected with a vector like pEGFP-N into cells, and then later the luciferase activity is determined to estimate the cleavage by the viral 3CL^pro^ [151]. Based on these reports, it seems that the isolated polyphenols displayed greater suppression on PL^pro^ than on 3CL^pro^ to exert their anti-SARS-CoV effects, suggesting that these compounds may be effective in inhibiting coronavirus replication.

**Figure 3:**
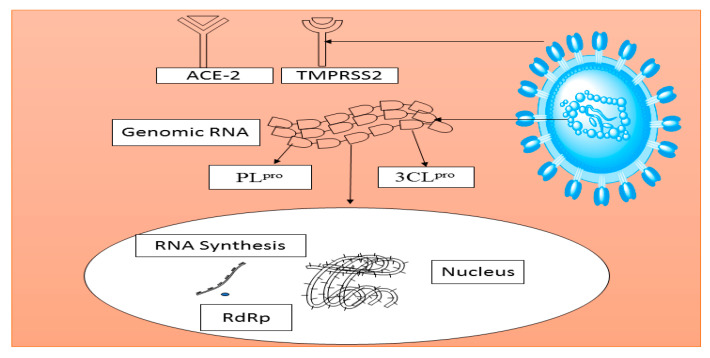
Schematic diagram of possible inhibitory sites against SARS-CoV that could be modulated by medicinal plants. ACE-2, angiotensin-converting enzyme 2; 3CL^pro^, 3C-like protease; PL^pro^, papain-like protease; RdRp, RNA-dependent RNA polymerase: TMPRSS2, transmembrane serine protease 2.

Moreover, many plant-based lectins have also been reported to demonstrate significant inhibitory activities against coronaviruses. Thirty-three lectins purified from different plant species were screened against SARS-CoV and feline coronavirus (FCoV). Mannose-binding lectin was identified to possess a notable anti-coronavirus activity by targeting the entry and the release of virus particles [167]. Agglutinin isolated from *Galanthus nivalis* was also reported to remarkably inhibit FCoV when administered in combination with nelfinavir, a synthetic antiretroviral drug [168]. These plant-derived constituents could be potential drug candidates for the treatment of SARS-CoV infection. Figure 3 depicts the possible specific target receptors and proteins in SARS-CoV that could be modulated by medicinal plants and their phytoconstituents. Another possible target is the type II transmembrane serine protease 2 (TMPRSS2). It can cleave the coronaviral receptor ACE-2 to amplify infectivity as well as activate the viral spike protein for membrane fusion [169]. Similar to DENV studies, no reports have been published regarding the antioxidant and anti-inflammatory effects of the medicinal plants on SARS-CoV infectivity, even though oxidative stress and inflammation have been demonstrated to play significant roles in the disease progression [170]. It is strongly believed that medicinal compounds with good antioxidant and anti-inflammatory properties might reduce or prevent the disease severity.

**Table 5:** Isolated compounds with anti-SARS-CoV activities.

Plant Source	Compounds	Mode of Action	Reference
*Boesenbergia. rotunda*	Panduratin	Inhibited SARS-CoV-2 in E6 cells	[38]
*Angelica keiskei*	Xanthoangelol	Inhibited PL^pro^ in vitro	[152]
*Alnus japonica*	Hirsutenone	Inhibited PL^pro^ in vitro	[166]
	Hirustanonol	Inhibited PL^pro^ in vitro	[166]
	Oregonin	Inhibited PL^pro^ in vitro	[166]
	Rubranol	Inhibited PL^pro^ in vitro	[166]
	Rubraanoside	Inhibited PL^pro^ in vitro	[166]
	Rubranoside A	Inhibited PL^pro^ in vitro	[166]
*Broussonetia papyrifera*	Broussochalcone B	Inhibited PL^pro^ better than 3CL^pro^	[153]
	Broussochalcone A	Inhibits PL^pro^ higher than 3CL^pro^	[153]
	4-Hydroxyisolonchocarpin	Inhibited PL^pro^ better than 3CL^pro^	[153]
	Kazinol	Inhibited PL^pro^ better than 3CL^pro^	[153]
	Kazinol A	Inhibited PL^pro^ better than 3CL^pro^	[153]
	Kazinol B	Inhibited PL^pro^ better than 3CL^pro^	[153]
	Kazinol F	Inhibited PL^pro^ better than 3CL^pro^	[153]
	Kazinol J	Inhibited PL^pro^ better than 3CL^pro^	[153]
	3′-(3-methylbut-2-enyl)-3′, 4,7-trihydroxyflavane	Inhibited PL^pro^ better than 3CL^pro^	[146]
	Papyriflavonol	Demonstrated highest inhibitory activity against PL^pro^ (IC_50_ = 3.7 µM)	[153]
*Cinnamomic verum*	Gallocatechin gallate	Inhibited wild-type SARS-CoV	[154]
*Euphoria nerifolia*	3ß-friedelanol	Exhibited potent antiviral activity against SARS-CoV higher than positive control	[149]
*Istatis indigotica*	Hesperetin	Inhibited 3CL^pro^ effect via cell free and cell-based cleavage assays	[151]
	Sinigrin	Inhibited 3CL^pro^ effect via cell free and cell-based cleavage assays	[151]
	Indigo	Inhibited 3CL^pro^ effect via cell free and cell-based cleavage assays	[151]
	Emodin	Inhibited 3CL^pro^ effect via cell free and cell-based cleavage assays	[142,155]

Abbreviations: 3CL^pro^, 3C-like protease; PL^pro^, papain-like protease; IC_50_, 50% of inhibitory concentration.

As previously mentioned, none of the mentioned studies was conducted in in vivo models. The potential of the anti-coronavirus activity of the extracts can be evaluated in many existing animal models like non-human primates, hamsters, mice, and ferrets [144], which are developed to ideally reflect the pathology and clinical signs of SARS-CoV in humans. Recently, a mouse model expressing human angiotensin-converting enzyme 2 (hACE-2) [165] and SARS-CoV-2−infected Syrian hamster model [171] were established to study the infection biology of SARS-CoV-2. Future studies can therefore employ these animal models to evaluate the efficacy of medicinal plants or pure compounds against SARS-CoV-2 for the development of antiviral or therapeutics.

## 6. Conclusions and Future Prospects

This review summarizes and updates various medicinal plants that are traditionally used to treat dengue and SARS-CoV-related infections, together with their pharmacological investigations against these viruses, using crude extracts or isolated/identified bioactive compounds. Many medicinal plants and pure compounds have shown promising activities even at clinical trials. However, many of the plants that are used traditionally for the infections are yet to be explored on their antiviral potentials. Extensive study is therefore needed to evaluate the potentials of these plants and their phytochemicals from in vitro to in vivo testing and then clinical studies.

Speedy identification of effective therapeutic measures against dengue and SARS-CoV has become a major challenge globally. Considering the fact that the development of new vaccines and synthetic drugs is highly time-consuming, it is therefore imperative to repurpose the alternative treatment or medications to suit the present situation. Alternative therapy from natural products seems to be capable of providing a valuable source for the swift discovery of antiviral drugs. The major challenge in the development of antiviral drugs that target specific viral proteins is the ability of the viruses to undergo rapid mutation during replication. Therefore, several aspects must be considered when evaluating the anti-dengue and anti-SARS-CoV-2 properties of medicinal herbs and pure compounds in terms of preparations, solvents, and techniques used for extraction. Many potent medicinal herbs reported here used polar solvents, which have been reported to aid in the attainment of the highest level of antiviral activity.

**Figure 4:**
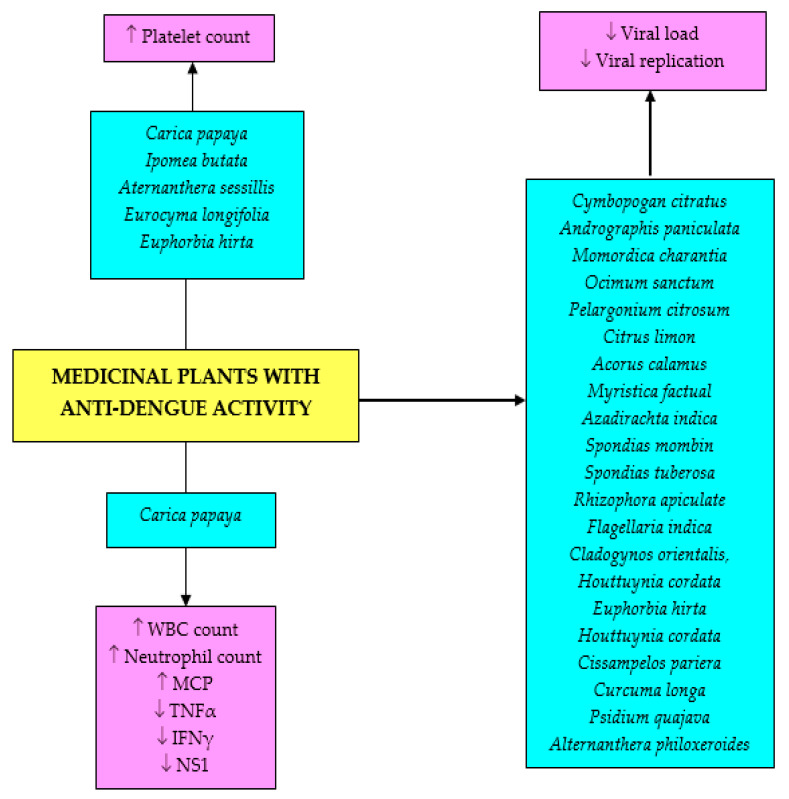
The effects of crude plant extracts on DENV infection. WBC, white blood cells; MCP, plasma monocyte chemoattractant protein; NS1, nonstructural protein 1; TNFα, tumor necrosis factor-α.

**Figure 5:**
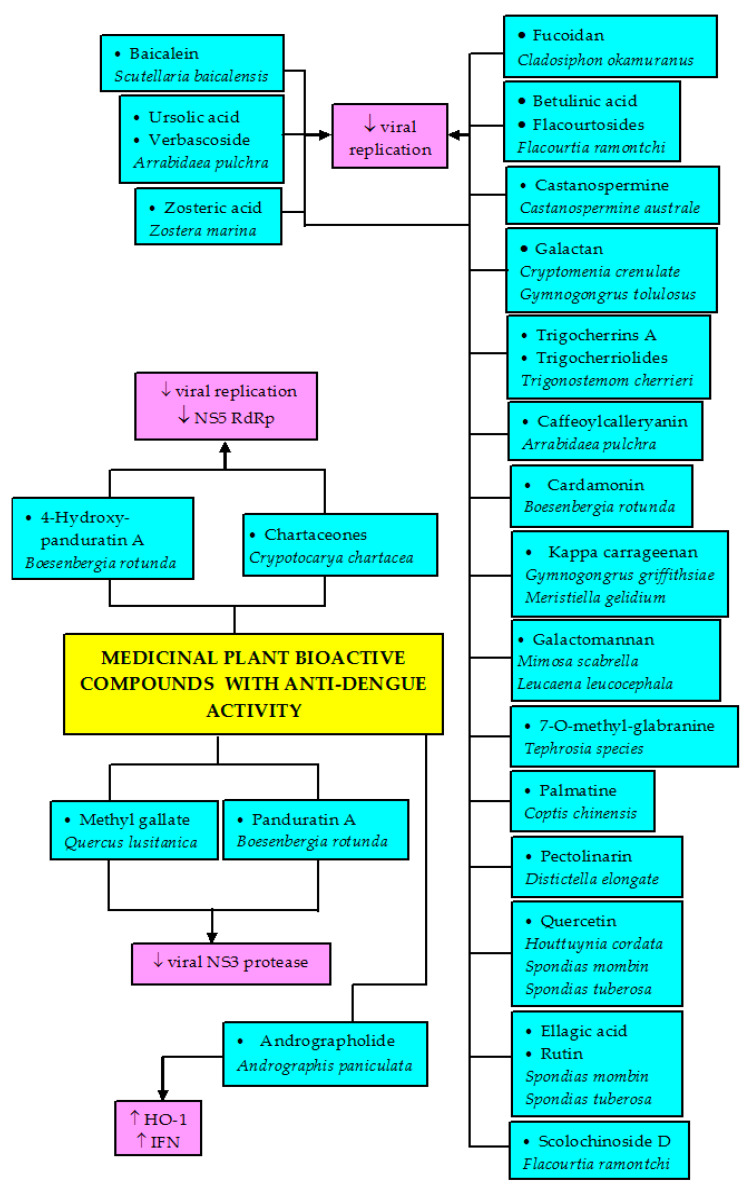
The effects of bioactive compounds isolated from plants with activity DENV infection. HO-1, heme oxygenase-1; IFN, interferon; NS5 RdRp, non-structural protein 5 RNA-dependent RNA polymerase; NS3, non-structural protein 3.

**Figure 6:**
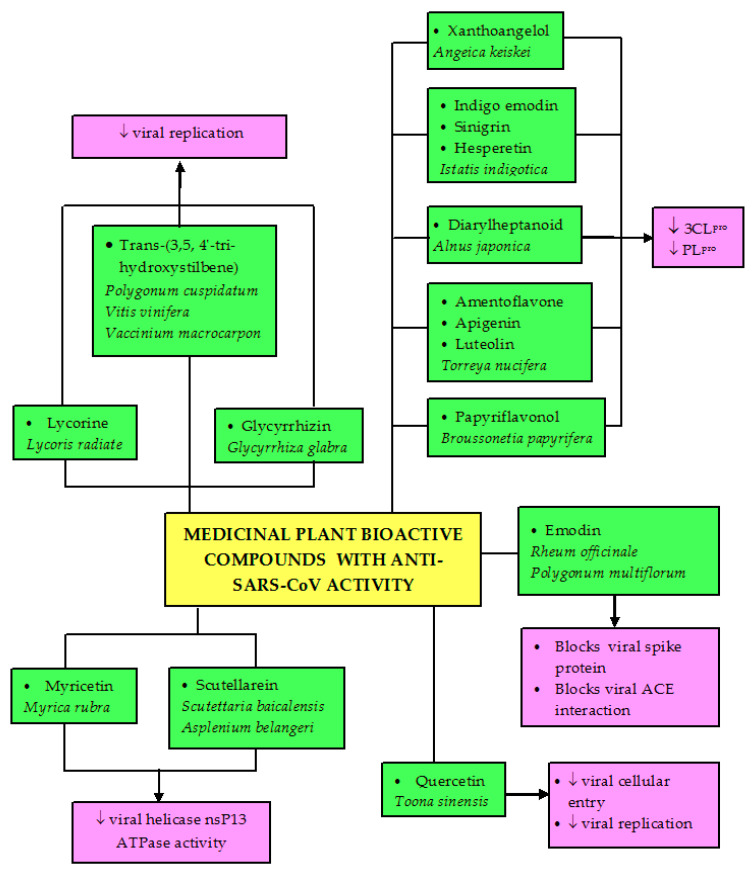
The effects of bioactive compounds isolated from plants with activity against SARS-CoV infection. ACE, angiotensin-converting enzyme; ATPase, adenosine triphosphatase; 3CL^pro^, 3C-like protease; PL^pro^, papain-like protease.

Even though a great number of research studies on anti-DENV and anti-SARS-CoV are still at initial stages, additional research is still very much required on the identification of the bioactive components, elucidation of the mechanisms of actions, evaluation of efficacy, and possible in vivo applications to promote the exploration of potent antiviral chemotherapeutics against these infections. Not much research has been done to study any potential immunomodulatory effects of the extracts or compounds, or their effects on signaling mechanisms leading to viral eradication. Additional investigations are also needed on the possibility of combining the existing treatment with natural bioactive compounds as a multiple-target solution to reduce the potentials of infection from drug-resistant virus strains. Moreover, there is a need for the standardization of herbal medicines to enhance high reliability, effectiveness, quality, and safety, which will require a series of scientific experiments ranging from the isolation of the active major compounds to different biological testing; pharmacokinetics, toxicological, and clinical studies. It is hoped that natural remedies such as medicinal plants and pure bioactive metabolites will play a significant role in the development and advancement of anti-dengue and anti-coronavirus drugs that are safe, efficacious, and cost-effective. The effects of the medicinal plant crude extracts and bioactive compounds on DENV infection are summarized in Figure 4 and Figure 5, while the effects of bioactive compounds on SARS-CoV infection is depicted in Figure 6.

## Figures and Tables

**Table 1:** Ethnomedicinal plants for the treatment of dengue fever.

Name	Family	Part Used	Preparation	Reference
*Annona reticulata*	Annonaceae	Barks and bulbs	Decoction	[60]
*Andrapogon citratum*	Poaceae	Oil	Oil put on candles	[66]
*Azadirachta indica*	Meliaceae	Leaves	Extracts	[64]
*Brassica campestris*	Brassicaceae	Oil	Mixture with camphor	[67]
*Carica papaya*	Caricaceae	Leaves	Juice	[56]
		Fruits	Oral decoction of unripe fruit	[59]
		Leaves and fruits	Not mentioned	[58]
		Female leaves	Decoction, (boiling the leaf juice)	[57]
		Leaves	Pounding and extraction	[55]
*Catharanthus roseus*	Apocynaceae	Whole plant	Oral decoction	[68]
*Citrus limon*	Rutaceae	Pulps	Juice mixed with *Protium spruceanum*	[63]
*Clerodendrum viscosum*	Lamiaceae	Leaves	Not mentioned	[66]
*Coleus scutellarioides*	Lamiaceae	Leaves	Juice	[60]
*Enicostemma hyssopifolium*	Gentianaceae	Not stated	Decoction	[69]
*Euodia species*	Rutaceae	Leaves	Water extract	[65]
*Euphorbia hirta*	Euphorbiaceae	Leaves	Decoction	[56,60]
		Leaves and stems	Decoction	[70]
		Roots	Oral decoction	[71]
		Leaves	Decoction and infusion	[61]
		Leaves, stems and roots	Decoction	[55]
*Mentha arvensis*	Lamiaceae	Leaves	Decoction	[60]
*Mikania micrantha*	Asteraceae	Leaves	Cold maceration	[72]
*Mikania cordata*	Asteraceae	Leaves	Leaves juice	[62]
*Musa paradisica*	Musaceae	Latex of the stems	Decoction	
*Ocinum sanctum*	Lamiaceae	Leaves	Juice	[57]
		Leaves	Juice	[67]
*Physalis angulata*	Solanaceae	Leaves	Oral infusion	[72]
*Phytolacca bogotensis*	Phytolaccaceae	Fresh leaves	Infusion of fresh leaves	[73]
*Protium spruceanum*	Burseraceae	Stem barks	Decoction with *Citrus limon*	[63]
*Psidium guajava*	Myrtaceae	Leaves	Decoction	[74]
		Leaves and young fruits	Not mentioned	[75]
*Tinospora cordifolia*	Menispermaceae	Leaves and stems	Decoction	[74]
*Trigonella foenam*	Fabaceae	Leaves	Water infusion	[74]
*Synespalum dulcificum*	Sapotaceae	Leaves	Juice	[60]
*Vitex negundo*	Lamiaceae	Leaves	Decoction	[60]
*Zingiber purpureum*	Zingiberaceae	Rhizomes and leaves	Oral decoction mixed with turmeric and onion	[65]

**Table 2:** Pharmacologically active medicinal plants for the treatment of dengue viral infection.

Plant	Family	Part Used	Type of Study	Mode of Action	Reference
*Alternanthera photoperiods*	Amaranthaceae	Whole plant	In vitro	Inhibited DENV-2 replication (IC_50_ = 47.43 µg/mL)	[89]
*Alternanthera sessillis*	Amaranthaceae	Leaves	In vivo	Significant platelet increasing activity	[77]
*Acorus calamus*	Acoracea	Leaves	In vitro	Inhibited DENV-2 replication (96.5% at a dose of 20 µg/mL)	[86]
*Andrographis paniculata*	Flaviviridae	Leaves	In vitro	Anti-DENV-1 activity in HEPG2 (78.3 ± 2.9 PFU/mL)	[88]
*Ocimum sanctum*	Lamiaceae	Leaves	In vitro	Anti-DENV-1 activity in HEPG2 (1020.0 ± 271.0 PFU/mL)	[98]
*Arrabidaea pulchra*	Bignoniaceae	Leaves	In vitro	Inhibited DENV-2 (EC_50_ = 46.8 ± 1.6 µg mL^−1^)	[84]
*Azadirachta indica*	Meliaceae.	Leaf extract	In vitro and in vivo	Inhibited DENV-2 replication in both in vitro and in vivo	[81]
*Carica papaya*	Caricaceae	Leaves	In vivo	Increased the platelet counts	[99]
		Leaf extract	Human	Increased the platelet counts and the total white cell counts had increased	[92]
		Leaf extract	Human	Increased the platelet counts	[91]
		Leaf extract	In vivo	Increased the platelet counts	[77]
		Leaf juice	Human	Increased the platelet counts	[93]
		Leaves	In vivo	Increased the platelet count and decreased the clotting time in rats	[76]
		Leaf juice	Human	Increased the platelet counts	[95]
		Leaf juice	In vivo	Increased the platelet counts	[78]
		Leaf juice	Human	Increased the platelet counts	[94]
		Leaves	In vivo	Increased the plasma CCL2/MCP-1 level	[82]
		Leaves	Human	Increased the platelet counts	[96]
*Cissampelos pareira*	Menispermacea	Aerial part	Human	Increased the platelet counts	[100]
*Cladogynos orientalis*	Euphorbiaceae	Whole plant	In vitro	Inhibited DENV-1–4 replication	[87]
*Curcuma longa*	Zingiberacea	Not stated	In vitro	Anti-DENV activity in Huh7it-1 cells (IC_50_ 17.91 = μg/mL)	[80]
			in vivo	Anti- DENV-2 and reduce viremia period	
*Cymbopogon citratus*	Poaceae	Root	In vitro	Inhibited DENV-2 replication (98.9% at a dose of 20 µg/mL)	[86]
*Distictella elongata*	Bignoniaceae	Leaves, stems and fruits	In vitro	Anti-DENV-2 activity (EC_50_ = 9.8 µg/mL)	[101]
*Euphorbia hirta*	Euphorbiaceae	Leaves	In vivo	DENV-2 inhibition by 34.7%	[15]
		Whole plant	In vivo	Increased the platelet counts	[77]
		Whole plant	In vitro	Significantly reduced the plaque forming capacity of the DENV-1-2 (85% and 90% respectively)	[90]
		Leaves	In vitro	Inhibited DENV-2 replication (34.7% at 20 µg/mL dose)	[102]
		Whole weed	Human	Increased the platelet counts	[97]
*Eurycoma longifolia*	Simaroubaceae	Root	In vitro	Inhibited DENV-1, DENV-2, DENV-3 and DENV-4 (IC_50_ = 33.84, 33.55, 58.35 and 119 µg/mL, respectively)	[79]
			In vivo	30% lower viral load and 12% higher platelet count compared to the control group	
*Flacourtia ramontchi*	Salicaceae	Stem barks	In vitro	Inhibited DENV-2 NS5 polymerase activity (89% inhibition at 10 μg/mL)	[103]
*Flagellaria indica*	Flagellariaceae	Whole plant	In vitro	Inhibited DENV-2 (45.52% at a dose of 12.5 µg/mL)	[87]
*Faramea bahinensis*	Rubiaceae	Leaves	In vitro	Anti-DENV activity in HEPG2 (100% reduction in viral load)	[104]
*Faramea hyacinthina* and *Faramea truncata*	Rubiaceae	Leaves	In vitro	Anti-DENV activity in HEPG2 (90 to 100% at a dose of 50 μg/mL)	[105]
*Faramea bahinensis*, *Faramea hyacinthina* and *Faramea truncata*	Rubiaceae	Leaves and stem	In vitro	Anti-DENV activity in HEPG2 (70 to 93% at a dose of 50 μg/mL)	[106]
*Hippophae rhamnoides*	Elaeagnaceae	Leaves	Human	Anti-DENV activity in BHK-21 cells (1 PFU/mL at a dose of 50 mg/mL), decreased TNF-α and increased IFN-*_Υ_*	[107]
*Houttuynia cordata*	Saururaceae	Whole plant	In vitro	Inhibited DENV-2 replication (35.99% at a dose of 1.56 µg/mL)	[87]
		Aerial stem and leaves	In vitro	Inhibited the intracellular viral RNA replication (EC_50_ = 0.8 µg/mL)	[36]
		Aerial leaves	In vitro	Inhibited DENV-2 replication (IC_50_ = 0.98 mg/µL)	[108,109]
*Ipomea batata*	Convolvulaceae	Leaves	In vivo	Significant platelet increasing activity	[77]
*Justicia adhatoda*	Acanthaceae		In vitro	Inhibited the growth of Vero cells infected with DENV-2 at a dose of 60 µg/m	[110]
*Piper retrofractum*	Piperaceae	Whole plant	In vitro	Inhibited DENV-2 replication (84.93%at a dose of 100 µg/mL)	[87]
*Psidium guajava*	Myrtaceae	Bark	In vitro	Inhibited DENV-2 replication	[111]
		Leaves	In vitro	Inhibited DENV-2 replication at a dose of 60 µg/µL	[110]
*Phyllanthus sp*	Phyllanthaceae	Whole plant	In vitro	Inhibited DENV-2 replication (91.48% at a dose of 250 µg/mL)	[49]
*Myristica fragrans*	Myristicaceae	Leaves	In vitro	Inhibited DENV-2 replication (122.7% at a dose of 20 µg/mL)	[86]
*Quersus lucitanica*	Fagaceae	Seed	In vitro	Inhibited DENV-2 (100% at a dose of 0.032 mg/mL)	[112]
*Rhizophora apiculata*	Rhizophoraceae	Whole plant	In vitro	Inhibited DENV-2 replication (56.14% at a dose of 12.5 µg/mL)	[87]
*Spondias mombin &*	Anacardiaceae	Leaves juice	In vitro	Inhibited DENV-2 replication (3.31% at a dose of 500 µg/µL)	[85]
*Spondias tuberosa*	Anacardiaceae	Leaves juice	In vitro	Inhibited DENV-2 replication (99% at a dose of 500 17.98 µg/mL)	[85]
*Uncaria tomentosa*	Rubiaceae	Stem barks of	In vitro	Reducing DENV-Ag+ cell rates	[113]
*Annona muricata*	Annonaceae	Fruit	In vitro	Inhibited DENV-2 replication (99% at a dose of 1.25 mg/mL)	[114]
*Catharanthus roseus*	Apocynaceae	Leaves	In vitro	Inhibited DENV-2 replication (60% at a dose of 0.078 mg/mL)	[115]
*Cynometra cauliflora*	Fabaceae	Leaves	In vitro	Inhibited DENV-2 replication (78% at a dose of 12.5 mg/mL)	[116]
*Orthosiphon stamineus*	Lamiaceae	Leaves	In vitro	Inhibited DENV-2 replication (88% at a dose of 0.31 mg/mL)	[117]

Abbreviations: EC_50_, median effective concentration; IC_50_, median inhibitory concentration; DENV, dengue virus; PFU, plaque forming units.

**Table 3:** Isolated compound with bioactivity against DENV infection.

Compound	Class of Compound	Plant Source	Antiviral Effects	Reference
Andrographolide	Diterpenoid	*Andrographis paniculata*	Anti-DENV activity in HepG2 (EC_50_ = 21.304 µM) and HeLa cell lines (EC_50_ = 22.739 µM)	[118]
			Good anti-DENV effects in both visual (EC_50_ = 0.56 µg/mL) and neutral red cytopathic effects (EC_50_ = 0.58 µg/mL)	[129]
			Reduced mean number of *A. aegypti* eggs and increased larvae mortality concentration-dependently	[119]
			Delayed disease onset, reduced mortality and DENV-1, DENV-2, DENV-3 and DENV-4 loads in infected mouse brains	[130]
Apiofuranoside	Flavanone glycosides	*Faramea bahiensis*	Controlled viral replication and reduced numbers of infected cells (12%) and RNA copies of DENV-2 (67%) in HepG2 cells	[104]
Betulinic acid 3ß-caffeate	Phenyl terpenoid	*Flacourtia ramontchi*	Inhibited DENV replication (IC_50_ = 0.85 µM)	[103]
Caffeoylcalleryanin	Phenolic glycoside	*Arrabidaea pulchra*	Anti-DENV-2 activity (IC_50_ = 2.8 µg/mL)	[84]
Cardamonin	Phenolic	*Boesenbergia rotunda*	Non-competitive anti-DENV activity	[37]
Castanospermine	Alkaloid	*Castanospermine australe*	Inhibited secretion and infectivity in all DENV serotypes in vitro. Only inhibited DENV-2 in vivo and prevented mouse mortality.	[120]
Chartaceones 1	Dialkylated flavanone	*Crypotocarya chartacea*	Inhibited NS5 RdRp (IC_50_ = 14.8 µM)	[127]
Chartaceones 2			Inhibited NS5 RdRp (IC_50_ = 72.7 µM)	[127]
Chartaceones 3			Inhibited NS5 RdRp (IC_50_ = 4.2 µM)	[127]
Chartaceones 4			Inhibited NS5 RdRp (IC_50_ = 1.8 µM)	[127]
Chartaceones 5			Inhibited NS5 RdRp (IC_50_ = 2.9 µM)	[127]
Chartaceones 6			Inhibited NS5 RdRp (IC_50_ = 2.4 µM)	[127]
Chlorogenic acid	Phenolic acid	*Houttuynia cordata*	Synergistic antiviral effect of combined hyperoside and chlorogenic acid	[109]
Ellagic acid	Phenolic acid	*Spondias mombin* and *Spondias tuberosa*	Anti-DENV-2 (25%) at 500 µg/mL	[85]
Flacourtoside A	Phenolic glycoside	*Flacourtia ramontchi*	Inhibited DENV replication (IC_50_ = 9.3 µM)	[103]
Flacourtoside B			Inhibited DENV replication (IC_50_ = 71.1 µM)	
Flacourtoside C			Inhibited DENV replication (IC_50_ = 23.8 µM)	
Flacourtoside D			Inhibited DENV replication (IC_50_ = 35.5 µM)	
Flacourtoside E			Inhibited DENV replication (IC_50_ = 13.4 µM)	
Flacourtoside F			Inhibited DENV replication (IC_50_ = 39.8 µM)	
Fucoidan	Sulfated polysaccharide	*Cladosiphon okamuranus*	Inhibited DENV-2 Reduced infectivity by 20% at 10 µg/mL in BHK-21 cells	[121]
Galactan	Sulfated polysaccharide	*Cryptomenia crenulate*	Inhibited DENV-2 and DENV-3 replication in Vero cells (IC_50_ = 1.0 µg/mL), slight inhibitory effect against DENV-4, inactive against DENV-1	[122]
		*Gymnogongrus tolulosus*	Inhibited DENV-2 and DENV-3 replication in Vero cells (IC_50_ = 0.19–1.7 µg/mL)	[123]
Galactomannan	Polysaccharride	*Mimosa scabrella*	Reduced DENV-1 titer 100-fold in C6/36 cells at 347 mg/L	[131]
		*Leucaena leucocephala*	Reduced DENV-1 titer 100-fold in C6/36 cells at 37 mg/L	[131]
Glabranine	Flavanone	*Tephrosia species*	Anti-DENV (70% inhibition) at 25 µM	[132]
Hyperoside	Flavonol glycoside	*Houttuynia cordata*	Synergistic antiviral effect of combination of hyperoside and chlorogenic acid	[109]
4-Hydroxy-panduratin A	Phenolic derivative	*Boesenbergia rotunda*	Competitive inhibitory activity against DENV-2 NS3 protease (K_1_ = 21 µM)	[37]
Kappa carrageenan	Sulfated polysaccharide	*Gymnogongrus griffithsiae*	Selectivity inhibited DENV-2 (IC_50_ = 0.9 µg/mL), DENV-3 and DENV-4 replication. Inactive against DENV-1	[122]
		*Meristiella gelidium*	Anti-DENV-2 activity (IC_50_ between 0.14–1.6 µg/mL)	[125]
7-O-methyl-glabranine	Flavanone	*Tephrosia species*	Exhibited 70% inhibition on DENV in a plaque-forming assay at 25 µM	[132]
Methyl gallate	Phenolic acid	*Quercus lusitanica*	Inhibited DENV-2 protease (98%) at 0.3 mg/mL	[133]
Palmatine	Alkaloid	*Coptis chinensis*	Inhibited DENV replication (EC_50_ = 26.4 µmol/L)	[134]
Panduratin A	Phenolic derivative	*Boesenbergia rotunda*	Competitive inhibitory activity against DENV-2 NS3 protease (K_1_ = 25 µM)	[37]
Pectolinarin	Flavone	*Distictella elongate*	Good anti-DENV-2 effect (EC_50_ = 86.4 µg/mL)	[101]
Pinostrobin	Flavanone	*Boesenbergia rotunda*	Non-competitive DENV replication inhibitory activity	[37]
Quercetin	Flavonoid	*Houttuynia cordata*	Anti-DENV-2 effect at 500 µg/mL (IC_50_ = 176.76 µg/mL)	[108]
Quercetin	Flavonol	*Houttuynia cordata*	Synergistic antiviral effect of quercetirin and quercetin combination	[109]
		*Spondias mombin* and *Spondias tuberosa*	Inhibited viral replication (50%) of DENV-2 at 500 µg/mL	[85]
Quercetirin	Flavonoid glycoside	*Houttuynia cordata*	Exhibited less activity in an uncombined form compared to its mixture with quercetin (IC_50_ = 467.27 µg/mL)	[108]
		*Houttuynia cordata*	Synergistic antiviral effect of quercetirin and quercetin combination. greater antiviral effects	[109]
Rutin	Flavonol glycoside	*Spondias mombin* and *Spondias tuberosa*	Inhibited viral replication of DENV-2 at 500 µg/mL by 68.42%	[85]
Scolochinenoside D	Phenolic glycoside	*Flacourtia ramontchi*	Inhibited DENV replication (IC_50_ = 9.5 µM)	[103]
Trigocherrin A	Diterpenoid	*Trigonostemon cherrieri*	Inhibited DENV replication (IC_50_ = 12.7 µM)	[128]
Trigocherriolide B	Diterpenoid	*Trigonostemon cherrieri*	Inhibited DENV replication (IC_50_ = 3.1 µM)	[128]
Trigocherriolide C	Diterpenoid	*Trigonostemon cherrieri*	Inhibited DENV replication (IC_50_ = 16.0 µM)	[128]
Ursolic acid	Triterpenoid	*Arrabidaea pulchra*	Displayed anti-DENV-2 (IC_50_ = 3.2 µg/mL)	[84]
Verbascoside	Phenyl glycoside	*Arrabidaea pulchra*	Displayed anti-DENV-2 (IC_50_ = 3.4 µg/mL)	[84]
Baicalein	Bioflavonoid	*Scutellaria baicalensis*	Displayed anti-DENV-2 (IC_50_ = 1.55 μg/mL)	[135]
Zosteric acid	Phenolic acid	*Zostera marina*	Displayed anti-DENV-2 (IC_50_ = 2.3 mM)	[136]

Abbreviations: EC_50_, median effective concentration; IC_50_, median inhibitory concentration; DENV, dengue virus; DENV-1, dengue virus type-1; DENV-2, dengue virus type 2, DENV-3, dengue virus type 3, DENV-4, dengue virus type 4, RdRp, RNA-dependent RNA polymerase; NS3, nonstructural protein 3.

**Table 4:** Medicinal plants with anti-SARS-CoV activity.

Species	Family	Part Used	Type of Study	Mode of Action	Reference
*Boesenbergia rotunda*	Zingiberaceae	Rhizome ethanol extract	In vitro	Suppressed SARS-CoV-2 infectivity in Vero E6 cells	[38]
*Broussonetia papyrifera*	Moraceae	Roots	In vitro	Chloroform fraction inhibited viral replication	[153]
*Euphoria nerifolia*	Euphorbiaceae	Leaves	In vitro	Exhibited antiviral activity against SARS-CoV	[149]
*Toona sinensis*	Meliaceae	Tender leaf extract	In vitro	Inhibited SARS-CoV replication	[148]
*Gentiana scabra*	Gentianaceae	Rhizomes		Inhibited viral replication of SARS-CoV in Vero E6 cells	[150]
*Dioscorea batatas*	Dioscoreaceae	Tubers	In vitro	Inhibited viral replication of SARS-CoV in Vero E6 cells	[150]
*Cassia tora*	Caesalpinioideae	Dried seeds	In vitro	Inhibited viral replication of SARS-CoV in Vero E6 cells	[150]
*Taxillus chinensis*	Loranthaceae	Dried stems and leaves	In vitro	Inhibited viral replication of SARS-CoV in Vero E6 cells	[150]
*Cibotium barometz*	Cibotiaceae	Rhizomes	In vitro	Inhibited viral replication of SARS-CoV in Vero E6 cells	[150]
*Cinnamomi verum*	Lauraceae	Butanol fraction	In vitro	Inhibited wild type SARS-CoV	[154]
*Houttuynia cordata*	Saururaceae	Dried whole plant		Had antiviral activity against SARS-CoV via inhibition on 3CL^pro^ and RNA-dependent RNA polymerase of the virus	[39]
*Isatis* *Indigotica*	Cruciferae	Root water extract	In vitro	Inhibited the cleavage activity of SARS 3CL^pro^ enzyme	[151]

Abbreviations: SARS-CoV-2, severe acute respiratory syndrome coronavirus-2; SARS-CoV, severe acute respiratory syndrome coronavirus; 3CL^pro^, 3C-like protease.
